# The impact of nonresponse in different survey stages on the precision of prevalence estimates for multi-stage survey studies

**DOI:** 10.1186/s13104-021-05840-0

**Published:** 2021-11-24

**Authors:** Ming Ma, Sophie Rosenberg, Alexander M. Kaizer

**Affiliations:** 1 Community and Behavioral Health, Colorado School of Public Health, University of Colorado Anschutz Medical Campus, 13055 E 17th Pl., MS F542, Aurora, CO 80045 USA; 2 Department of Biostatistics & Informatics, Colorado School of Public Health, University of Colorado Anschutz Medical Campus, 13001 E. 17th Pl., Campus Box B119, Aurora, CO 80045 USA

**Keywords:** Nonresponse, Precision, Multi-stage survey study

## Abstract

**Objective:**

While it is known that nonresponse might produce biased results and impair the precision of results in survey research studies, the pattern of the impact on the precision of estimates due to the nonresponse in different survey stages is historically overlooked. Having this type of information is essential when creating recruitment plans. This study proposes to examine and compare the effect of nonresponse in different stages on the precision of prevalence estimates in multi-stage survey studies. Based on data from a state level survey, a simulation approach was used to generate datasets with different nonresponse rates in three stages. The margin of error was then compared between the datasets with nonresponse at three different survey stages for 12 outcomes.

**Results:**

At the same nonresponse rate, the mean margin of error was greater for the data with nonresponse at higher stages. Additionally, as the nonresponse rate increased, precision was more inflated within the data with higher stage nonresponse. This suggests that the effort used to recruit the primary sampling units is more crucial to improve the precision of estimates in multi-stage survey studies.

## Introduction

Survey studies are commonly conducted to collect data from a population of interest, such as the attitudes, opinions, or social and health behaviors of individuals in the targeted population. The prevalence of indicators can provide agencies with useful information for making evidence-based policies, implementing programs, and evaluating intervention programs. Additionally, the information obtained from a representative sample helps researchers identify and monitor trends in areas of interest in the larger population and supports further research plans. Thus, reporting estimates with sufficient precision is important because less certainty indicates an increased variability within the population estimates.

It has been recognized that nonresponse leads to reduced sample sizes that can potentially produce biased estimates [1] and impair the precision of estimates. Rarely have studies investigated and compared the change in the precision influenced by nonresponse from different sampling stages in multi-stage survey studies. For example, in a health survey study that proposes to sample a group of clinics and then a group of patients within each sampled clinic, nonresponse can happen at either stage of sampling. First stage nonresponse occurs when a sampled clinic refuses to participate, while second stage nonresponse occurs when sampled patients fail to complete or refuse to participate in the survey. Nonresponses in both stages could result in a reduced sample size and higher variance for estimates, however it is not clear whether the nonresponse occurring at different stages in the study have similar impacts on the precision of estimates. For instance, in both the case where 90% of clinics responded with a 70% patient response rate and where 70% of clinics responded with a 90% patient response rate, an overall response rate of 63% will be found, given the assumption that clinics have roughly equal size and participating rates. This does not necessarily indicate that the prevalence estimates produced by the two surveys will have similar precision. Further insight on the effect of nonresponse occurring in different sampling stages is warranted to advise effort allocation during each stage of recruitment for survey studies. For example, if first stage nonresponse is more likely to result in inflated variance, researchers need to be aware and consider a more strategic recruiting plan to improve the response rate of the primary sampling units. Historically, the potential for results to be biased has been the primary concern within survey studies. However, the precision of the estimator from a survey study is just as important and improving nonresponse across different survey stages can lead to improved precision.

Oftentimes survey studies employ techniques for complex survey design and the calculation of variance involves the “design effect” that also depends on multiple components [2, 3], and thus it may not be intuitive to compare the impact of different stage nonresponse on variance using mathematical derivation. Additionally, to adjust for nonresponse, large weights are commonly applied and could cause excessive weight variation [4], which is also not as easily demonstrated using formulas. For example, most statistical survey software use the Taylor series expansion [5, 6] to estimate the variance for multi-stage sampling designs. In this study, by using data from a statewide adolescence survey project, we explore a simulation approach to assess and compare the impact of first, second, and third stage nonresponse on the sampling variance. This simulation method has been used in previous studies that investigate research questions related to variance estimation for complex survey data [7, 8]. We expect the findings from the simulation studies will provide evidence to reinforce the existing knowledge base and to better understand the patterns in the estimate’s precision due to different stage nonresponse with varied response rates.

## Main text

### Methods

#### Healthy kids colorado survey (HKCS) [9]

HKCS is a biannual statewide survey on the health and well-being of young Coloradans. The methods of sampling and data analysis for HKCS are aligned with the Youth Risk Behavior Survey conducted by the Centers for Disease Control and Prevention [10] that has been administered on a two-year cycle since 1991. The 2019 HKCS high school dataset was used as our analytical baseline dataset due to relatively higher response rates (83.4% for schools, 82.1% for classes, and 71.1% for students). The survey was administered from September to December of 2019 and included over 120 questions in domains such as physical activity, nutrition, bullying, substance use, school and teacher connections, mental health, and sexual behaviors.

In the first stage, 199 high schools (primary sampling units) were systematically sampled from 21 health statistic regions (strata). Of those, 33 sampled high schools refused to participate resulting in a school nonresponse rate of 16.6%. In the second stage, four or more classes were selected within each participating school, and all the students within the sampled classes were recruited. Of 3634 sampled classes, 651 classes failed to participate (class level nonresponse rate was 17.9%). A total of 65,468 9–12th grade students were sampled. Of those, 18,931 failed to complete the surveys (third stage student nonresponse rate was 28.9%). Weights were constructed to account for the selection probability, nonresponse, and difference in demographic distribution between the sample and the population of Colorado’s high school students [11, 12]. Weighting factors included: school base weight ($W_{1}$); school nonresponse adjustment factor ($F_{1}$); classroom selection weight ($W_{2}$); classroom nonresponse factor ($F_{2}$); an adjustment factor that accounts for student nonresponse ($F_{3}$); a post-stratification factor that adjusts the difference between the sample and the population ($F_{4}$). The final weights are the products of base weights and adjustment factors (final weight = $W_{1} F_{1} \times W_{2} F_{2} F_{3} \times F_{4}$), with extreme weights trimmed.

#### Baseline dataset

The sample included 46,537 9–12th graders from 2983 classes of 166 high schools from 21 health statistic regions. Twelve survey questions across several domains and their constructed binary variables were included in the baseline dataset for illustration: (1) Been active 60 min on more than 5 + days past 7 days; (2) Had 1 + drinks past 30 days; (3) Ate breakfast on all of the past 7 days; (4) Been bullied at school in past 12 months; (5) Fought 1 + times in past 12 months; (6) Described grades as mostly A’s or B’s over past 12 months; (7) Used marijuana 1 + times in past 30 days; (8) Never/rarely wore seat belt; (9) Ever had sex; (10) Slept 8 + hours/average school night; (11) Smoked 1 + days in past 30 days; (12) Attempted suicide 1 + times in past 12 months. Weighted prevalence for those indicators based on the baseline state dataset ranged from slightly lower than 10% to higher than 70%.

#### Simulation of nonresponding schools, classes, and students

Simulation was used to generate datasets with nonresponding schools, classes, and students at different rates. For example, to simulate the first stage school nonresponses, rates of 5%, 10%, 20%, 30%, 40%, 50%, and 60% of schools were randomly dropped from the baseline dataset. The simulation was repeated 1000 times at each nonresponse rate and 7000 datasets with seven different school nonresponse rates were created. A pre-compiled macro program was applied to construct survey weights for each simulated dataset, with the nonresponse adjustment factor (*F*_*1*_*)* and post-stratification factor (*F*_*4*_) calculated so the sum of the weight in the simulated datasets was identical to the original baseline state dataset. Similar procedures were used to simulate the scenarios of second stage class nonresponse and third stage student nonresponse.

#### Statistical analysis

**Fig. 1:**
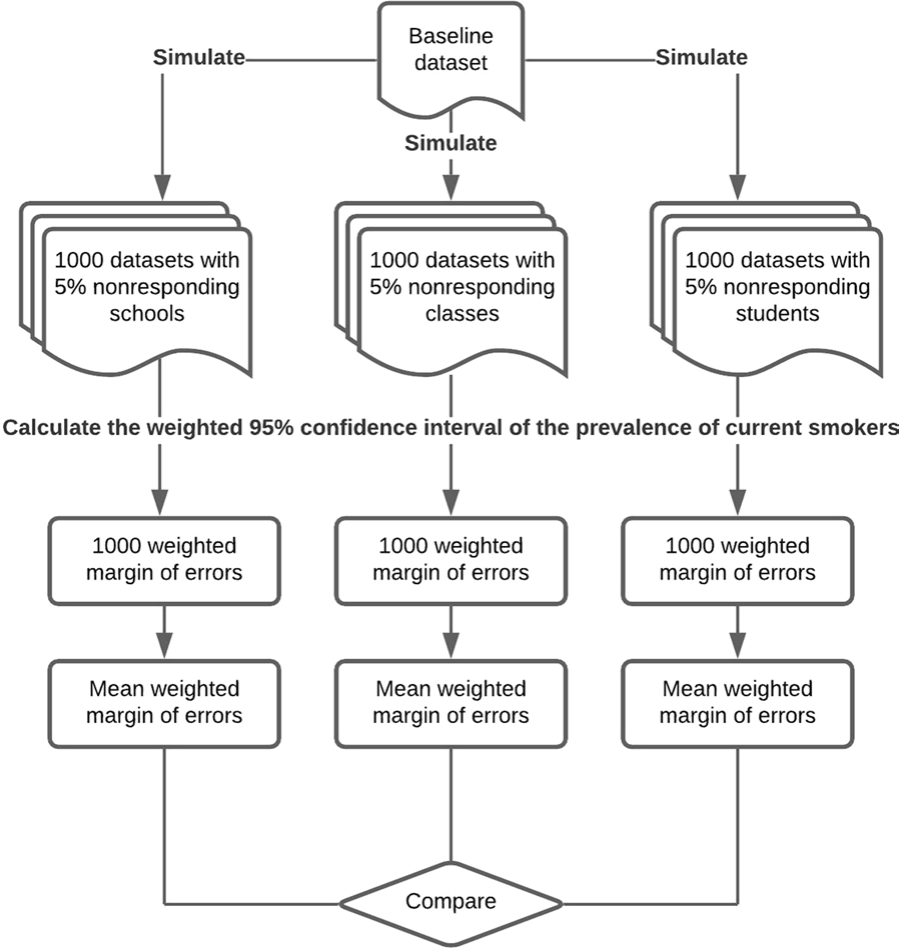
Flowchart of the simulation and data analysis procedure. This flowchart illustrates an example of the process to create 1000 datasets with 5% nonresponding schools, classes, and students, respectively, and the process to calculate and compare the mean margin of errors for the data with nonresponding schools, classes, and students, using current smokers as the survey outcome indicator. The simulation and data analysis were also conducted using other non-response rates (i.e., 10%, 20%, 30%, 40%, 50%, and 60%) and other survey question indicators

Weighted prevalence and the corresponding 95% confidence interval (CI) for the 12 binary outcome variables were estimated for each of the 21,000 simulated datasets. For each outcome variable, the mean margin of error of the point prevalence estimates at each nonresponse rate were calculated. For instance, to calculate the mean margin of error for the outcome variable “Attempted suicide” at a 10% school nonresponse rate, the margin of error was obtained from the half-width of each CI and then was averaged across the 1000 simulated datasets for that nonresponse rate. The means of each margin of error were plotted and compared at each nonresponse rate. Figure 1 is a flowchart to illustrates the simulation and data analysis procedure. The simulation and survey data analysis were all performed using SAS 9.4 (Cary, NC) [13].

### Results

The mean margin of error increased with increasing nonresponse rates for the simulated data with nonresponse at the first (school), second (class), and third (student) stages. However, at the same nonresponse rate, compared to the survey data with lower stage nonresponse (i.e., students/classes), the mean margin of error was greater for the data with higher stage nonresponse (i.e., nonresponding schools). Furthermore, with increasing nonresponse rates, the magnitude of the increase in the margin of error were more greatly inflated for the data with higher stage nonresponse.

**Fig. 2:**
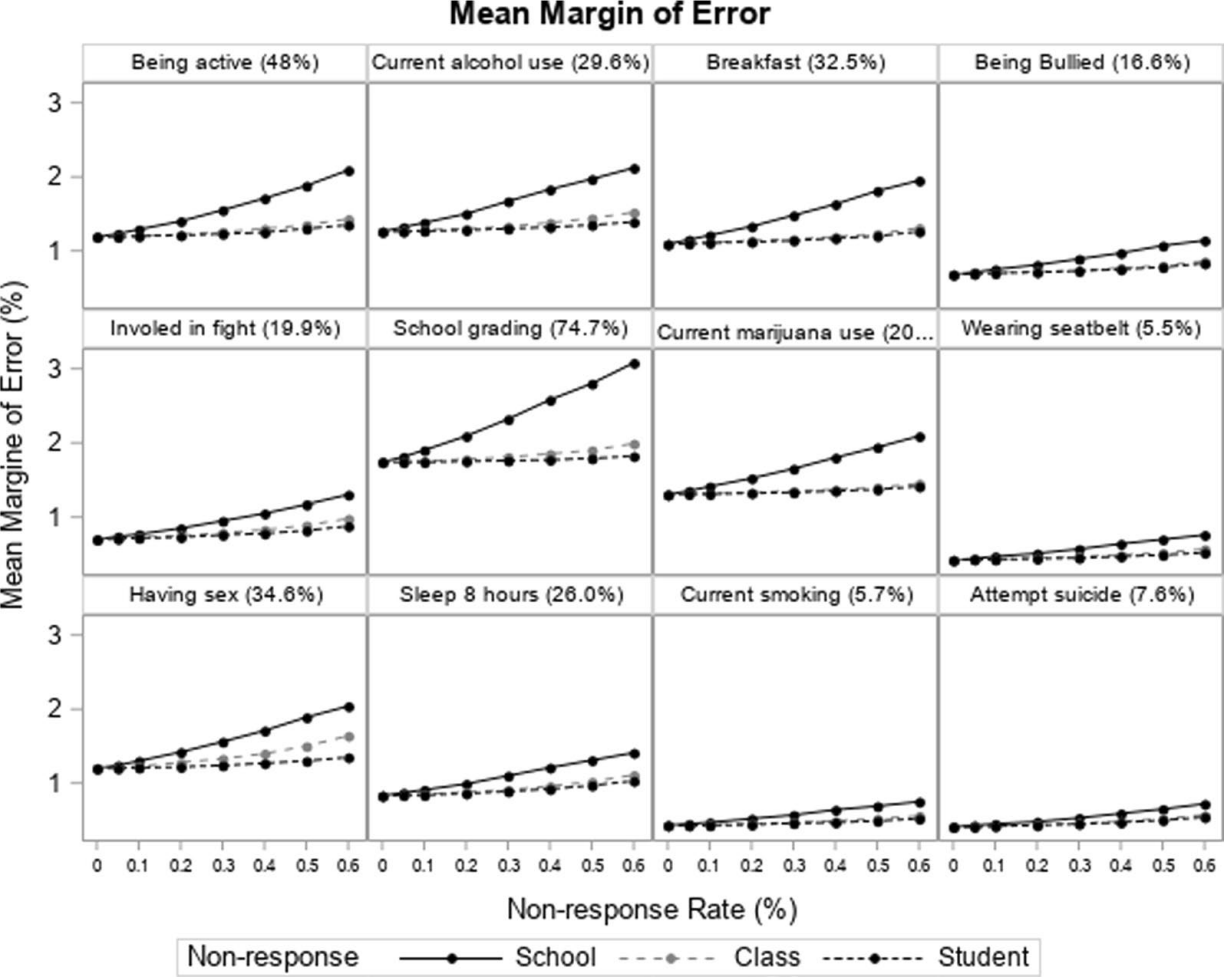
Mean margin of error for the estimates of the 12 outcomes at different school, class, and student non-response rates (The point estimate of the prevalence for the survey items were shown in the paratheses)

Because we randomly dropped schools, classes, and students from the baseline dataset, there was not substantial fluctuation in the point prevalence estimates for the data with simulated nonresponding schools, classes, and students across different nonresponse rates. However, the magnitude of the increase in the margin of errors were larger for the survey items with higher prevalence (i.e., school grading vs. current smoking). The mean margin of error for the 12 outcomes are shown in Fig. 2.

### Discussion

Although the adverse consequence of producing biased outcomes from nonresponse and the negative relationship between sample size and variance are both well known, the negative effect of nonresponse at different stages on variance estimation has not been thoroughly assessed. This study used a simulation approach to assess the impact that nonresponse at different sampling stages has on the variance estimation for survey data.

The findings from the simulation indicated that, under scenarios of identical nonresponse rates, higher stage nonresponse data was more likely to impair the precision of the estimates compared to lower stage nonresponse data. Furthermore, the magnitude in the difference of the variance was greater at higher nonresponse rates and was more pronounced for the survey items with higher prevalence estimates (i.e., school grading).

Our findings have reinforced existing knowledge regarding the variance of survey data. We further revealed that nonresponse at different sampling stages had different impact on the precision of estimates. The findings highlighted the need to place extra emphasis and resources on recruitment at higher sampling stages (e.g., primary sampling units), especially for survey studies with instruments that involve common or non-rare items. Though the simulation is based on the data from a single survey with categorical and ordinal responses, the evidence provided in this study can also be generalized to other multi-stage survey studies with similar structure and outcome types.

## Limitations

Some limitations of this simulation study need to be noted. First, it is often the case in real world survey research to have nonresponding sampling units in all the sampling stages. We simplified the scenario to investigate nonresponse at the first, second, and third stages separately, as it was not practical to simulate the scenarios with mixed nonresponse patterns. Second, because of the considerable amount of computation for generating 21,000 datasets, re-calculating weights, and data analysis, the replication of the simulation results may be challenging without access to adequate computational resources. Additionally, our simulation results focused on a single motivating survey with categorical and ordinal outcomes, but studies with different numbers of sampling stages or using continuous outcomes may have different results that also need to be verified in future research.

## Data Availability

The baseline dataset without school identifiers used in the simulation approach was 2019 HKCS Colorado Survey High School dataset which can be requested from Colorado Department of Public Health and Environment (CDPHE) (https://cdphe.colorado.gov/hkcs), the same dataset with school identifiers can be requested from the authors upon reasonable request and with permission of Colorado School of Public Health (CSPH). The SAS code used to create simulated datasets and analysis is available from the corresponding author (ming.ma@cuanschutz.edu) upon request.

## Abbreviations

HKCS: Healthy Kids Colorado Survey
*W*_1_: School base weight
*F*_1_: School nonresponse adjustment factor
*W*_2_: Classroom selection weight
*F*_2_: Classroom nonresponse factor
*F*_3_: Adjustment factor that accounts for student nonresponse
*F*_4_: Post-stratification factor that adjusts the difference between the sample and the population
